# Perception and Use of Compensation Strategies for Gait Impairment by Persons With Parkinson Disease

**DOI:** 10.1212/WNL.0000000000012633

**Published:** 2021-10-05

**Authors:** Anouk Tosserams, Lisanne Wit, Ingrid H.W.M. Sturkenboom, Maarten J. Nijkrake, Bastiaan R. Bloem, Jorik Nonnekes

**Affiliations:** From the Department of Neurology (A.T., B.R.B.) and Department of Rehabilitation (A.T., L.W., I.H.W.M.S., M.J.N., J.N.), Center of Expertise for Parkinson & Movement Disorders, Donders Institute for Brain, Cognition and Behaviour, Radboud University Medical Centre; and Department of Rehabilitation (J.N.), Sint Maartenskliniek, Nijmegen, the Netherlands.

## Abstract

**Background and Objectives:**

Gait impairments are common and disabling in Parkinson disease (PD). Applying compensation strategies helps to overcome these gait deficits. Clinical observations suggest that the efficacy of different compensation strategies varies depending on both individual patient characteristics and the context in which the strategies are applied. This has never been investigated systematically, hampering the ability of clinicians to provide a more personalized approach to gait rehabilitation. We had 3 aims: (1) to evaluate patients' awareness and actual use of compensation categories for gait impairments in PD, (2) to investigate the patient-rated efficacy of the various compensation strategies and whether this efficacy depends on the context in which the strategies are applied, and (3) to explore differences in the efficacy between subgroups based on sex, age, disease duration, freezing status, and ability to perform a dual task.

**Methods:**

A survey was conducted among 4,324 adults with PD and self-reported disabling gait impairments.

**Results:**

The main findings are as follows: (1) compensation strategies for gait impairments are commonly used by persons with PD, but their awareness of the full spectrum of available strategies is limited; (2) the patient-rated efficacy of compensation strategies is high but varies depending on the context in which they are applied; and (3) compensation strategies are useful for all types of patients with PD, but the efficacy of the different strategies varies per person.

**Discussion:**

The choice of compensation strategies for gait impairment in PD should be tailored to the individual patient and to the context in which the strategy needs to be applied.

**Classification of Evidence:**

This data provides Class IV evidence that compensation strategies are an effective treatment for gait impairment in patients with PD.

Gait impairments are common and are reckoned among the most disabling symptoms of Parkinson disease (PD). They often give rise to falls and fall-related injuries and decreased functional mobility, independence, and quality of life.^1-3^ Gait disturbances in PD can be continuously present (i.e., smaller step length, slower gait speed, or higher gait variability) or, as the disease progresses, become more episodic in nature (e.g., bouts of festination or freezing of gait [FOG]).^4,5^ Episodic gait deficits such as FOG may occur when the patient initiates gait, turns, or attempts to cross a narrow space (e.g., passing a doorway); when the patient is anxious; or when the patient performs a concurrent task while walking (e.g., talking or carrying a tray).^6-8^

Dopaminergic treatment alone is seldomly satisfactory in ameliorating these disabling gait impairments, especially with increasing disease duration.^9,10^ Remarkably, patients often spontaneously invent creative “detours” to overcome their walking difficulties in order to remain mobile and independent. These so-called compensation strategies can be very diverse; examples include walking paced by the rhythm of a metronome or by imaginary counting, mimicking the gait of another person, resorting to an adapted walking pattern (e.g., walking backward, lifting the knees up high), or using alternative ways to move forward such as roller skating. A wide range of different compensation strategies has been reported, typically in the form of anecdotal case reports, describing a typically self-invented solution that apparently worked very well for that particular individual.^11-13^ Recently, a comprehensive overview of compensation strategies to overcome gait impairments was published in which a conceptual framework of 7 separate overarching categories of compensation strategies was proposed based on their suspected underlying working mechanisms (Table 1).^14^

**Table 1:**
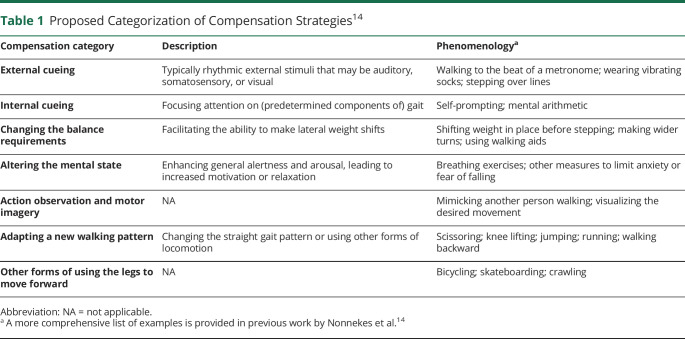
Proposed Categorization of Compensation Strategies^14^

Clinical observations suggest that a certain compensation strategy may be highly effective in one person but may have no effect on or even aggravate gait disability in another person. Furthermore, even within one individual, a specific strategy may have different effects depending on the context in which it is applied (e.g., when preparing food in the kitchen vs when walking outside).^15-17^ To date, this has not been systematically investigated, hampering the ability of health care professionals to provide a more tailored, personalized approach to gait rehabilitation for persons with PD. Consequently, in current daily practice, the search for appropriate compensation strategies for a given person with PD remains a time-consuming trial-and-error process. Moreover, individual patients are rarely offered an opportunity to systematically try out the multiple different variants of compensation strategies until they find a specific one that suits their needs and abilities best.

To address this issue, we conducted an international web-based survey among persons with PD experiencing gait impairments. The aim of this study was 3-fold: (1) to evaluate the participants' awareness and use of the various compensation categories for gait impairments in PD, (2) to investigate the patient-rated efficacy of compensation strategies and whether this depends on the context in which the strategies are applied, and (3) to explore whether different patient subgroups (defined by sex, age, disease duration, freezing status, and ability to perform a dual task) might respond differently to certain types of compensation strategies.

## Methods

### Study Design

The primary research questions were as follows. (1) To what extent do participants know and use the 7 categories of compensation strategies for gait impairments in PD? (2) What is the patient-rated efficacy of compensation strategies, and does this differ depending on the context in which the strategies are applied? (3) Do different subgroups of participants (defined by sex, age, disease duration, freezing status, and ability to perform a dual task) respond differently to certain categories of compensation strategies (Class IV evidence)?

A web-based survey was distributed among 6,700 participants within the Fox Insight cohort, as well as 1,573 Dutch participants via ParkinsonNEXT (the Netherlands). Fox Insight is a longitudinal, virtual, patient-centered observational study on PD led by the Michael J. Fox Foundation. Data used in the preparation of this article were obtained from the Fox Insight database on June 1, 2020. For up-to-date information on the study, interested readers should visit the Fox Insight website. ParkinsonNEXT is an online platform that aims to unite patients, researchers, and clinicians wanting to contribute to research and innovation in PD or parkinsonism. The online survey was accessible from March to June 2020. Respondents >18 years of age with a self-reported diagnosis of PD and self-reported disabling gait impairments were included in the analyses.

The survey consisted of 3 parts (eAppendix 1, links.lww.com/WNL/B492). The first part asked about sex, age, time since PD diagnosis, and the presence and severity of gait impairments. Moreover, the presence and severity of FOG were assessed with the New Freezing of Gait Questionnaire.^18^ They were also asked about their fall history over the preceding 12 months. The second part of the survey addressed the 7 main categories of compensation strategies (Table 1).^14^ One by one, each specific category was explained and illustrated by several practical examples. Participants were then queried whether they were aware of the category of strategies, whether they had ever applied a strategy belonging to that category, and, if so, how application of this strategy had affected their gait in a variety of contexts. These contexts included gait initiation, turning, stopping, passing a doorway, walking in narrow spaces, walking outdoors, walking in a crowded area, walking while talking, walking while carrying something, performing activities of daily living, and time-pressure situations. Respondents could indicate whether applying the strategy in that specific context improved their gait, had no effect on their gait, or worsened their gait. The third part of the survey examined the participants' interest to learn more about compensation strategies for gait impairments in PD. At the end of the survey, respondents were given an open-ended opportunity to share any new compensation strategies other than the ones already presented in the overview. In all cases, the mentioned strategies fitted into 1 of the 7 proposed categories and were therefore migrated to the corresponding categories.

For Fox Insight respondents, data from a preexisting Fox Insight questionnaire, Your Cognition and Daily Activities, were also included in the analyses. No data on cognition were available for respondents from the ParkinsonNEXT cohort.

### Data Processing and Analysis

According to the free-text entries that respondents had provided, data were verified and manually corrected by 2 independent researchers (A.T., L.W.) to ensure that all recorded compensation strategies were completed under the appropriate corresponding category. All (descriptive) statistical analyses were performed in IBM SPSS 25 (SPSS, Inc, Chicago, IL). Any missing values were excluded from the analyses. Independent *t* tests (means) and χ^2^ tests (proportions) were performed to assess subgroup differences. Values of *p* < 0.05 were considered to be statistically significant.

### Standard Protocol Approvals, Registrations, and Patient Consents

The study was approved by the Institutional Review Board of the Radboud University Medical Center in Nijmegen, the Netherlands (reference: 2019-5737). Written informed consent was not necessary for this work.

### Data Availability

Data are available to qualified investigators on request to the corresponding author.

## Results

### Study Population

In total, 4,987 responses were collected via Fox Insight (response rate 74.4%) and 845 via ParkinsonNEXT (response rate 53.7%). The 1,508 persons who did not report disabling gait impairments were excluded. Characteristics of the included sample of 4,324 respondents are presented in Table 2*.* Differences in the main characteristics of responders vs nonresponders from the ParkinsonNEXT cohort were not clinically relevant in terms of sex distribution (62.1% vs 64.6% men), age (66.4 years vs 64.5 years), and disease duration (6.5 years vs 5.9 years since diagnosis). These data were not available for the Fox Insight sample.

**Table 2:**
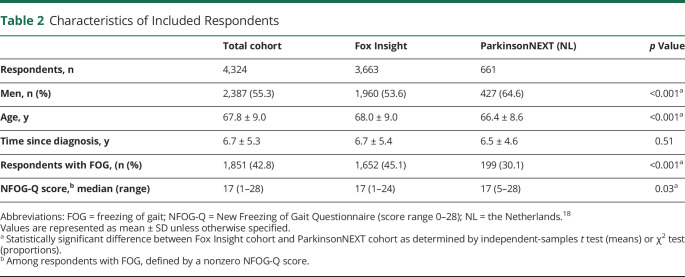
Characteristics of Included Respondents

Of the 4,324 persons with gait impairments who were included, 35.3% found that their walking difficulties negatively affected their ability to perform their usual daily activities. Of note, 52.4% of respondents had experienced ≥1 falls in the preceding 12 months, resulting in injury that had required medical attention in 385 cases.

Data from the Fox Insight questionnaire Your Cognition and Daily Activities were available for 3,586 of 3,663 (97.9%) respondents from the Fox Insight cohort enrolled in the present study. The majority of these respondents had little to no difficulties performing cognitive tasks in daily life. Specifically, most respondents had little to no difficulties reading the newspaper or a magazine (88.7%); keeping track of time (e.g., using a clock) (96.1%); counting the correct amount of money when making purchases (96.8%); reading or following complex instructions (e.g., directions for a new medication) (90.9%); handling an unfamiliar problem (e.g., getting the refrigerator fixed) (88.0%); explaining how to do something involving several steps to another person (84.0%); remembering a list of 4 or 5 errands without writing it down (69.1%); using a map to tell where to go (92.1%); remembering new information such as phone numbers or simple instructions (77.2%); doing >1 thing at a time (76.0%); learning to use new gadgets or machines around the house (84.2%); understanding their personal financial affairs (92.5%); maintaining or completing a train of thought (92.5%); discussing a TV show, a book, a movie, or current events (88.5%); or remembering what day and month it is (93.8%). Fewer than 1% of respondents indicated that they were completely incapable of performing ≥1 of these daily activities.

### Awareness of Compensation Strategies

Of all respondents, 16.7% had never heard of any of the compensation strategies before. Only a small group (3.5%) was aware of all 7 categories of compensation strategies. The median number of categories that respondents were aware of was 3. Apart from the use of walking aids and alternatives to walking, external cueing was the most widely known category of compensation strategies (46.9% had heard of it), followed by internal cueing (44.8%). Action observation and motor imagery was the least known category (14.3%). Dutch respondents from the ParkinsonNEXT cohort generally knew more categories of strategies (median 4) compared to respondents from within the Fox Insight cohort (median 3; *p* < 0.001).

Most respondents had read about the strategies themselves (35.0%), had heard about the strategy from their physical therapist (29.6%), or had invented the strategies themselves (12.5%). One in 3 participants (32.2%) had ever received targeted advice from a professional focused on the use of compensation strategies for gait impairments in PD. Notably, 75.2% of respondents indicated that they would be interested to learn more about the available compensation strategies.

### Use of Compensation Strategies

Of all respondents, 22.8% had never tried any form of compensation strategies before, despite experiencing clear and sometimes disabling gait impairments. Fewer than 1% of respondents had tried all 7 categories of compensation strategies. The median number of categories that respondents had ever tried was 2. Adapting a new walking pattern was tried most often (78.4% of respondents who were aware of it had ever tried it), followed by internal cueing (76.8%). Alternatives to walking was the least tried category (28.3%).

Overall, 64.7% of respondents still used ≥1 compensation strategies in daily life. Compensation strategies were most often used when walking outdoors or in time-pressure situations and were least often applied when attempting to stop walking or cross a doorway. The median number of categories used in daily life was 1. Changing the balance requirements was the most widely used category. Among the 1,729 users of this category, 429 (24.8%) respondents used walking aids only, whereas 1,300 (75.2%) also used other balance strategies (e.g., making a volitional weight shift to initiate gait). After changing the balance requirements, internal cueing was most often applied in daily life (71.7% of respondents who had tried it continued to use it), followed by altering the mental state (70.5%). External cueing was the least used category (55.3%).

Among the respondents using compensation strategies, 12.4% reported that they had felt obliged to switch to different strategies over time. Most often, this was due to PD progression, rendering some strategies too difficult or dangerous to apply (e.g., riding a bicycle). Another illustrative example included switching from walking over lines pasted to the floor to using a specialized Parkinson wheeled walker that is able to project a laser line on the floor. We found no suggestion that the effect of a certain strategy tapered off over time due to habituation.

### Patient-Reported Efficacy of Compensation Strategies

**Figure 1:**
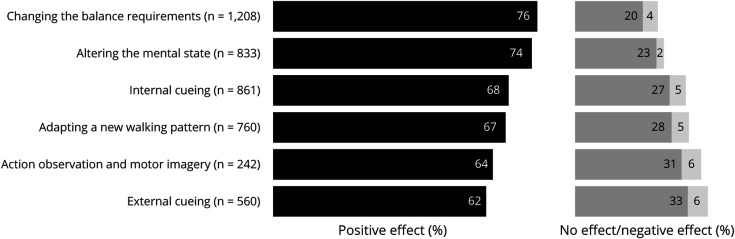
Overall Patient-Reported Efficacy of Different Compensation Strategies for Gait Impairments in Parkinson Disease Sample size represents the number of respondents who indicated that they had ever tried that specific category of strategies. Values represent the percentage of respondents experiencing a positive effect averaged across all contexts to provide an overall indication of efficacy.

The patient-reported efficacy of the different categories of compensation strategies is presented in Figure 1. While most respondents reported that the application of compensation strategies positively affected their gait, not every respondent seemed to benefit from every category of strategies. When the efficacy of the strategies was averaged across contexts, changing the balance requirements had the highest success rate in improving gait (76%), whereas external cueing showed the relatively lowest success rate (62%).

**Figure 2:**
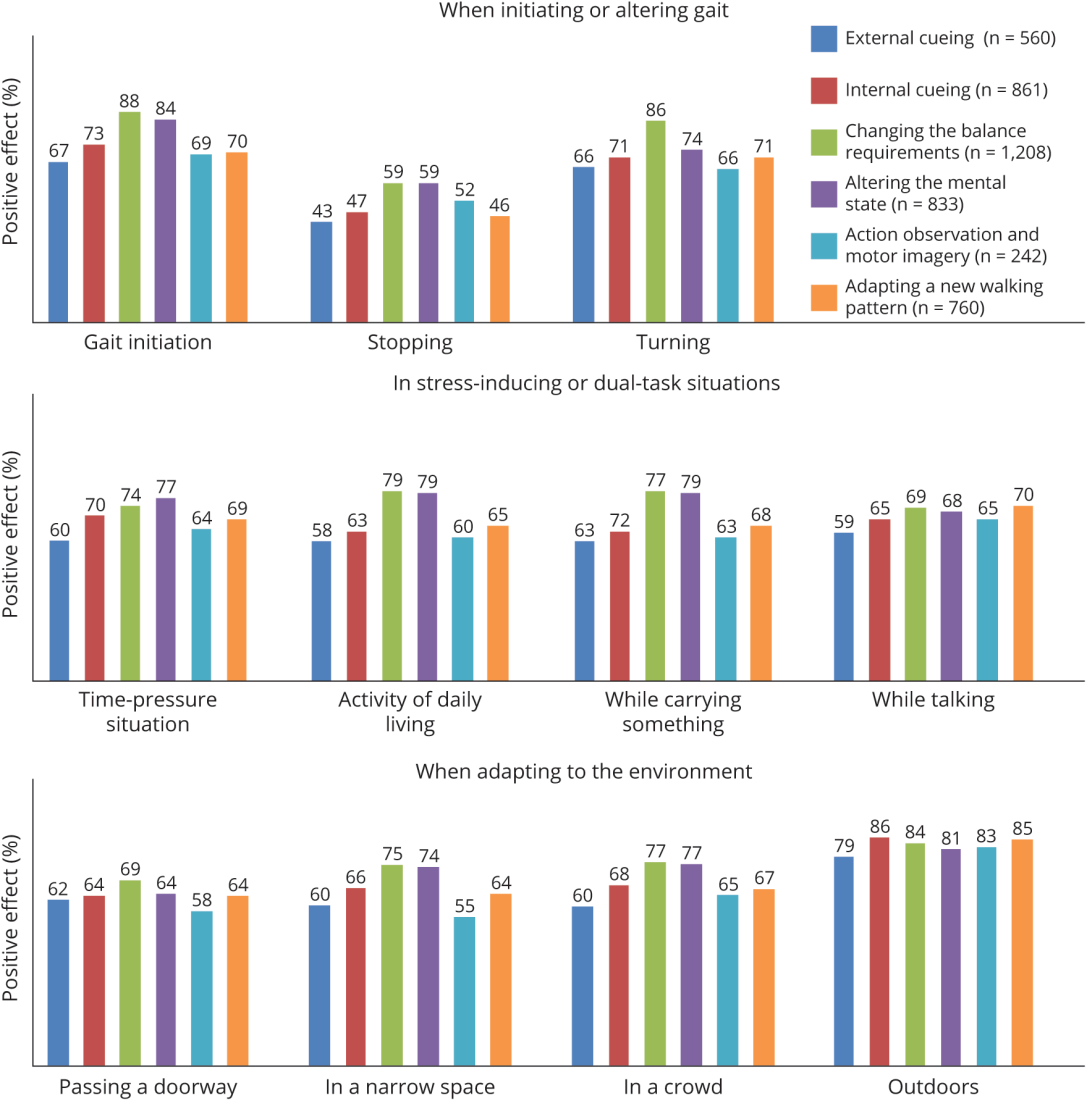
Patient-Reported Efficacy (Percent Indicating a Positive Effect) of Different Compensation Strategies for Gait Impairments in Parkinson Disease, Depending on the Context in Which They Were Applied Values represent the percentage of users experiencing a positive effect on gait impairments while applying a specific strategy in a specific context.

The efficacy of compensation strategies varied greatly, depending on the context in which they were applied (Figure 2). Internal cueing, for example, seemed highly effective during gait initiation (73% success rate) but was deemed to be less useful when attempting to stop walking (47%). Similarly, action observation and motor imagery could be a successful strategy when walking outdoors (83% success rate) but seemed to be less helpful when applied in a narrow space (55%). In general, compensation strategies were most effective when walking outdoors (84% success rate) or during gait initiation (79%). Strategies were deemed least effective during an attempt to stop walking (54% success rate) or cross a doorway (65%). While reports of negative effects of compensation strategies were relatively scarce (this occurred in ≈3% of cases), paradoxical aggravation of gait deficits was occasionally reported in stress-inducing or dual-task situations, including time-pressure situations (6%), walking in narrow spaces or crowded areas (7%), and walking while talking or carrying something (7%).

### Subgroup Data

The awareness and use of compensation strategies for gait impairments in PD did not differ between subgroups based on sex, freezing status, age (cutoff 65 years), time since diagnosis (cutoff 5 years), and the ability to perform a dual task (persons with little to no difficulties vs persons with more severe difficulties). There were also no differences in the reported efficacy of different strategies between these subgroups except for a slightly higher success rate among younger patients for external cueing and adopting a new walking pattern and among persons who had little to no difficulty dual tasking for motor imagery and action observation (Table 3).

**Table 3:**
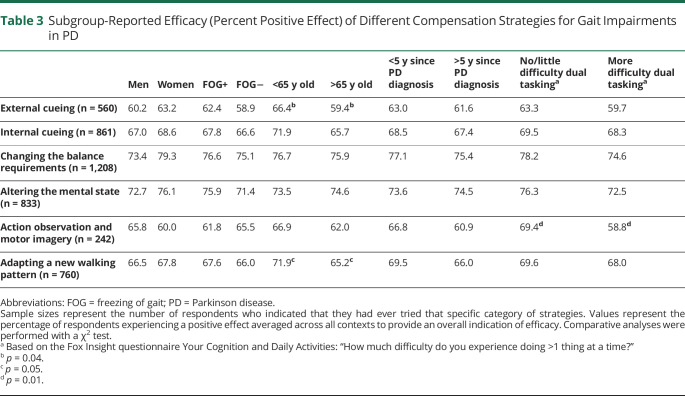
Subgroup-Reported Efficacy (Percent Positive Effect) of Different Compensation Strategies for Gait Impairments in PD

## Discussion

A web-based survey among 4,324 persons in the Fox Insight and ParkinsonNEXT (the Netherlands) cohorts was conducted to make an inventory of patients' perceptions of compensation strategies for gait impairments in PD. The main findings of this study were as follows: (1) compensation strategies are commonly used by persons with PD and gait impairments, although their awareness of the full spectrum of available strategies is limited; (2) the patient-rated efficacy of compensation strategies is high but varies depending on the context in which they are applied; and (3) the efficacy of compensation strategies varies per person, emphasizing the need for a more personalized approach to gait rehabilitation in PD. We discuss these findings in further detail below.

First, considering the severity of walking difficulties that respondents expressed, we consider the awareness of the full spectrum of compensation strategies among persons with PD to be rather limited. The median number of known categories was 3 of 7, and a striking 1 in 5 patients had no prior awareness of any of the compensation strategies for gait impairments. About half of the respondents had acquired this knowledge themselves through reading or personal experience. Notably, only 1 in 3 patients had ever received targeted advice from a professional, focused specifically on strategies to overcome gait impairments. Moreover, only 1% of patients had tried strategies from all 7 categories available. A previous study among health care professionals in the Netherlands demonstrated that only 23% of PD health care professionals (i.e., physiotherapists, occupational therapists) apply all 7 categories of compensation strategies in clinical practice when working with patients with PD experiencing gait impairments due to the lack of specific knowledge and skills in this field.^19^ Considering that PD care in the Netherlands is organized in a high-standard professional network of therapists who have received dedicated PD-specific training and treat large numbers of patients (ParkinsonNet),^20^ this percentage might be an overestimation of the global knowledge and application of compensation strategies among PD health care professionals. This may also explain why Dutch respondents from ParkinsonNEXT knew more strategies than respondents from Fox Insight.

Regardless of the underlying explanations, it is evident that both persons with PD and PD health care professionals^19^ are interested in learning more about compensation strategies. Integrating the use of compensation strategies into educational programs or developing a dedicated online platform about the various available strategies might facilitate finding a suitable strategy for every person with PD who experiences gait impairment. This notion is underscored by the present findings showing that the application of a single strategy is often insufficient because different contexts may require different types of strategies or because individual patients simply respond better to one specific strategy compared to another. In addition, our findings show that the feasibility of a previously successful strategy may diminish over time because of progression of disability, emphasizing the need to have a broader spectrum of compensation strategies available so that a customized renewed approach can be identified for any given individual patient. In the present work, we did not investigate whether knowing more compensation strategies positively affected a person's perceived quality of life, but this could be a topic of future investigations.

Second, the overall patient-rated efficacy of compensation strategies is high across all 7 categories. While the main body of scientific work on compensation strategies has thus far focused on external cueing, perhaps because external cueing is easily controllable in a laboratory setting,^21-24^ it is the least effective category according to patients. Unsurprisingly, because it is the most commonly known and applied category among PD health care professionals,^19^ the existence of external cueing was widely known among respondents. Yet, only few patients actually applied external cues in their daily lives. In contrast, strategies changing the balance requirements and altering the mental state were deemed to be most effective and were accordingly most often used. These categories may be more accessible and feasible for persons with PD because they typically do not require specific devices (e.g., laser shoes, a metronome) or adaptations to the environment (e.g., 2- or 3-dimensional patterns on the floor). They may also be preferred because they are relatively less noticeable to bystanders, avoiding stigmatization or feelings of embarrassment.^25,26^ Therefore, our findings reinforce the notion that all categories of compensation, not just external cueing, deserve further systematic investigation.

The effects of compensation strategies vary depending on the context in which they are applied, underlining the importance of a tailored approach to gait rehabilitation. We were struck by the relatively modest effect of compensations strategies during attempts to stop walking. More work is needed to clarify why gait termination is less influenced by the application of compensation strategies and what alternative strategies could be developed to ameliorate this.

Another notable finding was that some respondents experienced a negative effect of compensation strategies during stress-inducing or dual-task situations. In PD, gait deficits are generally exacerbated while dual tasks are performed because the need to concentrate on executing the concurrent task interferes with the patient's ability to focus purposefully on gait.^27-29^ Compensation strategies are believed to aid in prioritizing tasks and in allocating attention to gait.^14^ However, the introduction of an additional task, namely the application of a compensation strategy, might exceed the attentional resources in certain individuals, causing a paradoxical aggravation of walking difficulties instead of an improvement.^17^ This is also reflected in the present study by the slightly higher efficacy ratings of respondents with little to no difficulties with dual tasking compared to respondents with more difficulties with dual tasking.

Third, the efficacy of compensation strategies varies per person. Exploratory subgroup examinations based on age, sex, freezing status, and disease duration, however, did not demonstrate truly remarkable differences in the patient-rated efficacy of the different categories of compensation strategies. In general, compensation strategies seem to be useful in all types of patients with PD, and further work is needed to investigate optimal predictors of the effects of the different types of strategies for individual patients. Ideally, this would be investigated in a prospective clinical trial in which a clinician could also examine the severity of gait impairments present and the efficacy of the different strategies could be quantified with the use of more objective measures (e.g., improvement of spatiotemporal gait parameters, including gait variability).

Our study was not without shortcomings, and some of our findings should be interpreted with caution. Participants of both cohorts may well have been a selected and rather proactive sample of the overall PD population. Although the response rate was high for a survey study, particularly in the Fox Insight cohort, respondents may have been the most informed or motivated persons with PD. In addition, we were unable to retrieve information on the main characteristics of nonresponders from within the Fox Insight sample. The self-reported nature of the survey is further reason for caution, for example, because the extent of the gait disability could not be confirmed by an independent neurologic examination. Because PD diagnosis is also self-reported, we cannot exclude the possibility that some patients in fact had a form of atypical parkinsonism, which may respond in a different way to compensation strategies than PD. At the same time, it is quite possible that persons with atypical parkinsonism will also benefit from compensation strategies, and such compensation may be particularly important for these patients because medication is generally much less effective in improving their gait impairments. Another limitation of our study is the lack of objective information about the respondents' cognitive status; cognitive deficits may impede the ability to use particular categories of compensation strategies.^17,30^ It is possible that a certain degree of cognitive reserve is imperative to be able to compensate for gait impairments.^31^ The potential effect of impaired cognition might be particularly relevant for compensation strategies that are inherently cognitive tasks such as internal cueing (e.g., counting while walking). Further studies should aim to include a more heterogeneous study population in terms of cognitive status and include more objective measures of cognition (e.g., the Mini Mental State Examination or Montreal Cognitive Assessment) to gain more insight into the interplay between cognition and the ability to compensate for gait impairment in PD.

The present findings support the application of compensation strategies for gait impairments in PD and emphasize that a one-size-fits-all approach to gait rehabilitation is inappropriate. Persons with PD should be—and wish to be—more thoroughly informed about the range of available strategies. The choice of compensation strategies should be tailored to the individual patient and to the contexts in which the strategies need to be applied. Further prospective studies are vital to further crystallize these findings and eventually incorporate them into evidence-based protocols, thus paving the way toward a more personalized approach to gait rehabilitation in PD.

## Glossary

FOG: freezing of gait
PD: Parkinson disease
